# Supplementary material for: Development of an Efficient Protocol to Obtain Transgenic Coffee, Coffea arabica L., Expressing the Cry10Aa Toxin of Bacillus thuringiensis
Source: Int J Mol Sci. 2019 Oct 26;20(21):5334. doi: 10.3390/ijms20215334 (PMC6862211; doi:10.3390/ijms20215334)
Supplement: Supplementary file 1 [file ijms-20-05334-s001.pdf]

```
Or-atgaatccatatcaaaataagaatgaatatgaaatattcaatgctccatccaatggttttagcaagtctaataactattctagatatccattagcaaataag
Op-atgaaCccCtaCcaaaaCaagaatgaataCgaaatTttcaatgcAccatccaaCggCtttagcaagAGCaataactaCtcCagataCccattGgcaaataag
Aa- M N P Y Q N K N E Y E I F N A P S N G F S K S N N Y S R Y P L A N K

Or-ccaaatcaaccactgaaaaacacgaattacaaagattggctcaatgtgtgtcaagataatcaacaatatggcaataatgcggggaattttgctagttctgaa
Op-ccaaatcaaccactCaaaaacacTaattacaaagaCtggctTaatgtTgtcaagataaCcaacaatatggcaataatgcAggTaattttgctagttctgaa
Aa- P N Q P L K N T N Y K D W L N V C Q D N Q Q Y G N N A G N F A S S E

Or-actattgttggagttagtgcaggtattattgtagtaggaactatgttaggagcttttgctgcccctgtcttagctgcaggtataatatctttgggactttg
Op-actattgttggagttagCgcaggtattattgtTgtTggaactatgttGggTgcttttgctgcAcctgtTCtCgctgcTggAataatTAGCtttggCactttg
Aa- T I V G V S A G I I V V G T M L G A F A A P V L A A G I I S F G T L

Or-ttgccgatcttttggcaaggatctgaccctgcaaatgtttggcaggatttgttaaacatcggaggaaggcctatacaagaaatagataaaaacataattaat
Op-CttccAatTttCtggcaaggTtcCgacccAgcTaatgtttggcaggatCtTttGaacatTggaggaaggcctatTcaagaaatTgaCaaGaacatTattaat
Aa- L P I F W Q G S D P A N V W Q D L L N I G G R P I Q E I D K N I I N

Or-gtactaacttctatcgtaacacctataaaaaatcaacttgataaatatcaagaatttttcgataaatgggagccagcacgtacacacgctaatgctaaagca
Op-gtTctTactAGCatTgtTacTccAataaaaaatcaacttgaCaaatatcaagaatttttcgaCaaatgggagccagcaAgGacacacgctaatgcAaaGgcT
Aa- V L T S I V T P I K N Q L D K Y Q E F F D K W E P A R T H A N A K A

Or-gtacatgatctctttactaccttagaacctataatagataaagatttagatatgttaaaaaataatgctagctatcgaataccaacactccctgcatatgca
Op-gtTcatgaCTtGtttacCacACtTgaaccAatTatTgataaagaCCtTgaCatgCtCaaaaataatgctTCAtatcgaatTccaacTTtGccAgcatatgcT
Aa- V H D L F T T L E P I I D K D L D M L K N N A S Y R I P T L P A Y A

Or-caaatagctacttggcacttgaatttattaaaacatgctgctacctattacaatatatggctgcaaaatcaaggtataaatccaagtactttcaattcatct
Op-caaatTgctacttggcaTttgaatCtCCtCaaacatgctgctacTtattacaatatTtggTtgcaaaatcaaggtatTaatccaTCCactttTaaCtcCtcC
Aa- Q I A T W H L N L L K H A A T Y Y N I W L Q N Q G I N P S T F N S S

Or-aattactatcagggctatttaaaacgtaaaatacaagaatatactgactattgtatacaaacgtacaatgcaggactaactatgattagaactaatactaac
Op-aaCtactatcaAggTtatCtTaaaAgGaaGatTcaagaatatactgactattgtatTcaaacTtacaatgcaggactaactatgattagaactaatactaac
Aa- N Y Y Q G Y L K R K I Q E Y T D Y C I Q T Y N A G L T M I R T N T N

Or-gcaacatggaatatgtataatacttaccgtttagaaatgactctaactgtgttagatcttattgctatttttccaaattatgacccagaaaaatatccaata
Op-gcaacatggaatatgtataatacttacAgAttagaaatgactctTactgtTttGgaCcttattgctatttttccaaattatgacccagaaaaatatccaatT
Aa- A T W N M Y N T Y R L E M T L T V L D L I A I F P N Y D P E K Y P I

Or-ggagttaaatctgaacttatcagagaagtttatacgaatgttaattcagatacatttagaaccataacagaactagaaaatggattaactagaaatcctaca
Op-ggagttaaatcAgaacttatTCgagaagtttatacTaatgttaatAGCgatacTtttagaacTatTacagaactTgaaaatggaCtTactagaaatccAaca
Aa- G V K S E L I R E V Y T N V N S D T F R T I T E L E N G L T R N P T

Or-ttatttacttggataaaccaagggcgtttttacacaagaaattctcgagacattcttgatccttatgatatttttctttacaggtaaccagatggccttt
Op-ttGtttacttggatTaaTcaaggTAgAttCtacacaagaaattcAAgagacattcttgatccAtatgatatttttctttacaggtaaccagatggccttt
Aa- L F T W I N Q G R F Y T R N S R D I L D P Y D I F S F T G N Q M A F

Or-acacatactaatgatgatcgcaacataatctggggagcggttcatggaaatattatttctcaagacacatccaaagtatttccttttatagaaacaaacct
Op-acacatactaatgatgatAgAaacatTatTggggCgcTgttcatggTaatattatttcCcaagaTacatccaaagtTttccAtttatagaaacaaaccA
Aa- T H T N D D R N I I W G A V H G N I I S Q D T S K V F P F Y R N K P

Or-attgataaggtcgaaattgtcagacatagagagtactcagatataatatatgaaatgatatttttttcgaatagcagtgaagtatttcgatattcatccaat
Op-attgaCaaggtcgaaattgtcagacatagagagtactcCgaCatTatTtatgaaatgatTttCttCAGCaaCagcTCCgaagtTttcgTtatAGCtcAaat
Aa- I D K V E I V R H R E Y S D I I Y E M I F F S N S S E V F R Y S S N

Or-tcaacaatagaaaataattataaaagaactgattcttatatgattccaaaacaaacatggaaaaataaagaatatggtcatactctatcgtatataaaaact
Op-tcCacTatTgaaaataattataaaagaactgaCAGttatatgattccaaaacaaacatggaaaaataaagaatatggtcatactctTtcCtatataaaaact
Aa- S N S T I E N N Y K R T D S Y M I P K Q T W K E Y G H T L S V I K T

Or-gataattatatattttcagtagttagagaaagaagaagagttgcatttagttggacacatactagtgttgatttccaaaatacaatagatttagataacatc
Op-gaCaattatatTtttAGCgtCgttagagaaagaagaagagttgcatttTCCtggacacatactagCgttgaCttccaaaatacaatTgaCCtTgaCaacatT
Aa- D N V I F S V V R E R R R V A F S W T H T S V D F Q N T I D L D N I

Or-acccaaatccacgctctaaaagctttgaaggtaagttctgattcgaaaattgtgaaaggtcctggtcacacaggtggagacttggtaattcttaaagatagt
Op-acTcaaatTcacgctctTaaagctttgaaggtTTCCtcCgaCtcCaaaattgtTaaGggCccAggtcacacTggtggagacttggtTattcttaaagatTCC
Aa- T Q I H A L K A L K V S S D S K I V K G P G H T G G D L V I L K D S

Or-atggatttagagttagattttaaaaaatgtttctcgacaatatcaagtacgtattcgttatgctactaatgctccaaagacaacagtattcttaaccgga
Op-atggaCtttagagttagattttGaaaaatgtttctAgacaatatcaagtTAgAattAgAtatgctactaatgctccaaagacaacagtTttcCtTacTgga
Aa- M D F R V R F L K N V S R Q V Q V R I R Y A T N A P K T T V F L T G

Or-atagatactataagtgtggagctccctagtaccacttcccgccaaaacccaaatgctacagatttaacatatgcagattttggatatgtaacatttccaaga
Op-atTgatactatTTCCgtTgagctTccATCCacAacttccAgAcaaaacccaaatgctacagatttGacatatgcTgaCtttggatatgtaacatttccaaga
Aa- I D T I S V E L P S T T S R Q N P N A T D L T Y A D F G Y V T F P R

Or-acagttccaaataaaacatttgaaggagaagacactttattaatgaccttatatggtacaccaaatcattcatataatatatatattgacaaaatcgaattt
Op-acagttccaaataaaacTtttgaaggagaagacactCtTCtTatgacTttGtatggtacaccaaatcattcCtataatatTtatattgacaaaatcgaattt
Aa- T V P N K T F E G E D T L L M T L Y G T P N H S Y N I Y I D K I E F

Or-attccaatcactcaatctgtattagattatacagagaagcaaattatagaaaaaacacagaaaatagtgaatgatttatttgttaat
Op-attccaatcactcaatcCgtTCtTgaCtatacTgaAaagcaaaAtatTgaGaaGacTcaAaaGatCgtCaatgaCCtTttCgtGaat
Aa- I P I T Q S V L D Y T E K Q N I E K T Q K I V N D L F V N
```

Supplementary figure 1. Original (Or) and optimized (Op) sequences of the *cry10Aa* gene from *B. thuringiensis* serovar *israelensis,* along with their corresponding amino acid (Aa) sequence. Changes in the optimized nucleotide sequence are shown in capital letters.

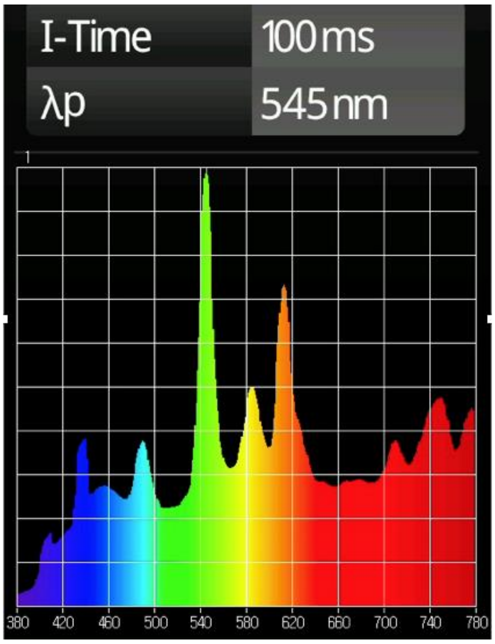

Supplementary figure 2. Light spectrum used for maturation of somatic embryos and plant regeneration of coffee, *C. arabica*.
